# Neutrophil to lymphocyte ratio (NTLR) predicts local control and overall survival after stereotactic body radiotherapy (SBRT) in metastatic sarcoma

**DOI:** 10.1038/s41598-023-46476-3

**Published:** 2023-11-07

**Authors:** Eashwar Somasundaram, Peter M. Anderson, Timothy D. Smile, Ahmed Halima, James B. Broughman, Chandana A. Reddy, Shireen Parsai, Jacob G. Scott, Timothy Chan, Shauna Campbell, Lilyana Angelov, Stacey Zahler, Matteo Trucco, Stefanie M. Thomas, Shavaughn Johnson, Peng Qi, Anthony Magnelli, Erin S. Murphy

**Affiliations:** 1 Case Western Reserve University School of Medicine, Cleveland, OH USA; 2 Department of Radiation Oncology, Taussig Cancer Center, Cleveland Clinic, R3 9500 Euclid Ave, Cleveland, 44195 OH USA; 3 Department of Radiation Oncology, Ohio Health Riverside Methodist Hospital, Columbus, OH USA; 4 Rose Ella Burkhardt Brain Tumor and Neuro-Oncology Center, Cleveland Clinic, Cleveland, OH USA; 5 Department of Pediatric Hematology Oncology and Blood and Marrow Transplantation, Cleveland Clinic, Cleveland, OH USA

**Keywords:** Cancer, Oncology

## Abstract

The neutrophil to lymphocyte ratio (NTLR) and absolute lymphocyte count (ALC) recovery are prognostic across many cancers. We investigated whether NLTR predicts SBRT success or survival in a metastatic sarcoma cohort treated with SBRT from 2014 and 2020 (N = 42). Wilcox Signed Rank Test and Friedman Test compare NTLR changes with local failure vs. local control (N = 138 lesions). Cox analyses identified factors associated with overall survival. If local control was successful, NLTR change was not significant (*p* = 0.30). However, NLTR significantly changed in patients with local failure (*p* = 0.027). The multivariable Cox model demonstrated higher NLTR before SBRT was associated with worse overall survival (*p* = 0.002). The optimal NTLR cut point was 5 (Youden index: 0.418). One-year overall survival in SBRT metastatic sarcoma cohort was 47.6% (CI 34.3%–66.1%). Patients with an NTLR above 5 had a one-year overall survival of 37.7% (21.4%–66.3%); patients with an NTLR below 5 had a significantly improved overall survival of 63% (43.3%–91.6%, *p* = 0.014). Since NTLR at the time of SBRT was significantly associated with local control success and overall survival in metastatic sarcoma treated with SBRT, future efforts to reduce tumor inhibitory microenvironment factors and improve lymphocyte recovery should be investigated.

## Introduction

Sarcomas are a group of rare mesenchymal tumors that account for less than 1% of adult solid malignancies, but represent 21% of pediatric solid malignancies^1^. While the incidence is low relative to other malignancies, their biological heterogeneity and relative treatment resistance have made sarcomas challenging to treat^2^. Sarcomas can be divided into soft tissue sarcomas and primary bone tumors (e.g. osteosarcoma and Ewing sarcoma). Advances in sarcoma staging and therapy have led to survival improvements in both classes of sarcomas as well as improved risk-stratification^3^. For example, the 5-year survival in rhabdomyosarcoma has improved from around 50 to 70%^4^. Advancements in chemotherapy have improved prognosis in patients with low grade tumors. However, the success of surgery, radiotherapy, and chemotherapy capable of treating advanced metastatic disease is low^5,6^. Many patients present with high-grade histopathological features, and up to 25% of patients present with distant metastasis^3,4^. The current standard of care for most sarcomas is a combination of chemotherapy and local control that includes surgical resection and/or radiotherapy. Radiation is typically reserved for unresectable disease, when surgical morbidity could limit function, or when metastases are present^7^. There is an unmet need for more data to improve quality of cancer care and survival^8^.

Stereotactic body radiation (SBRT) is a relatively new advancement in radiotherapy that uses image guidance to precisely deliver high doses of ablative radiation in fewer fractions compared to traditional dose regimens. While contemporary indications for SBRT are rapidly expanding, it is most commonly used to treat inoperable early stage non-small cell lung cancer, prostate cancer, unresectable pancreatic cancer, pulmonary and hepatic metastases, and metastases to the spine or brain via a stereotactic radiosurgery approach^9^. Some sarcomas are relatively radioresistant and would theoretically benefit from the high biologically equivalent doses (BED) accompanying SBRT regimens. A multi-institutional phase II trial showed a 95% rate of lesion-specific local control at 6 months and improved progression free survival (PFS) and OS in patients with unresectable metastatic sarcoma treated with SBRT^10^. While local control with SBRT is encouraging, careful consideration of the therapeutic window must be applied as the higher fractional doses of radiation from SBRT increase the risk of developing late complications in normal tissue. A retrospective analysis of 31 patients found similarly high rates of local control and minimal toxicity, with only 1 out of 31 patients experiencing a late grade 3 radiation related toxicity^11^. SBRT has typically been reserved as salvage therapy for treatment resistant disease, so its role in primary treatment remains unknown, but has been investigated on a large cohort in a completed Children’s Oncology Group clinical trial in patients with metastatic Ewing sarcoma (NCT02306161).

Radiation therapy induces DNA damage through direct interaction and generation of reactive oxygen species. Tumor cells that survive the direct toxicity can express new antigens that may be recognized by the host immune system^16^. After radiation induced cell death of cancer cells, neutrophils and lymphocytes are actively recruited to the tumor site^17^. Given the interactions between radiation and the immune system, there may be a promising role for immunotherapy and radiotherapy synergism in the treatment of malignancy. SBRT, rather than conventional radiotherapy, may especially be synergistic with the patient’s immune system because it causes less lymphocyte depletion^18^. In pediatric osteosarcoma and Ewing sarcoma, improved absolute lymphocyte count (from either lymphocyte resilience or earlier recovery of lymphocytes post chemotherapy) has demonstrated survival benefits^19–22^. If the immune system is a critical part of the radiation efficacy, it would be helpful to have biometrics that help characterize the state of one’s immune system prior to SBRT.

Currently, there is no gold standard biomarker or biometric that can be used to assess how a patient’s immune system may respond to radiation. The NTLR is a promising candidate that has been correlated with survival in many malignancies including sarcoma before surgery^23^ and after chemotherapy^24^. Elevated pretreatment NTLR have been associated with poorer prognosis in both solid and hematological malignancies^25–28^. In soft tissue sarcoma, it has been associated with poorer survival and increased risk of distant metastasis^29^. Lymphopenia is also a prognostic factor in advanced sarcomas^30^.

While the literature suggests that NTLR will likely be prognostic across cancer diagnoses, it has not been studied in this population of metastatic sarcoma patients treated with SBRT. In this study, we show how NTLR changes over the SBRT treatment course and optimize a NTLR cutoff score to stratify patients with metastatic sarcoma into high and low risk survival groups. Furthermore, we demonstrate high NTLR was associated with a significantly worse prognosis and local failure after SBRT in patients with metastatic sarcoma.

## Methods

### Cohort

This study was approved by the Cleveland Clinic Institutional Review Board (IRB) as a single-institution registry of patients with metastatic sarcoma diagnosed between 2014 and 2020 treated with SBRT. After discussion of indications, risks, benefits, and alternatives, all patients or legal guardians provided written informed consent for SBRT as part of standard clinical care. No additional blood samples were obtained, therefore this was not considered an interventional study requiring additional informed consent for additional research blood samples as per IRB guidelines for a retrospective study. All research was performed in accordance with relevant guidelines and regulations. Included patients had histologically confirmed sarcoma including clear cell sarcoma, desmoplastic small round cell tumor, Ewing sarcoma, myxoid liposarcoma, osteosarcoma, rhabdomyosarcoma, undifferentiated pleomorphic small round blue cell sarcoma, and synovial sarcoma with at least one metastatic lesion treated with SBRT. CBC with differential data was collected by retrospective chart review at time points prior to SBRT, after SBRT, in follow-up, and at time of progression.

### Treatment

Patients with histopathologic diagnosis of sarcoma were treated with SBRT defined as a fractional dose of ≥ 5 Gy delivered in five or fewer fractions to site(s) of metastatic disease. The intent of therapy -usually either definitive for oligo-metastatic disease or palliative for durable local control of metastases—as well as the specific dose and fractionation regimen were determined at the discretion of the treating physician. Technical details of SBRT technique based on lesion location have been described previously^11,31^.

### Laboratory investigations

For each lesion treated with SBRT, complete blood count (CBC) data before and after SBRT treatment and in follow up were recorded. Since CBC is not routinely measured immediately before or after treatment, we included values within 3 months of treatment. We also recorded CBC parameters collected within 3 months after any local failure. We calculated a neutrophil to lymphocyte ratio by dividing absolute neutrophil count by absolute lymphocyte count. Friedman Test was used to assess changes in NTLR through SBRT therapy in failed lesions (comparing before SBRT vs after SBRT vs after local recurrence). Wilcox signed rank was used to compare NTLR in locally controlled lesions before SBRT versus after SBRT. These nonparametric statistical methods were chosen over linear regression methods due to the non-normality of the data and limited time points for comparison of the dependent variables.

### Imaging

Patients received routine post-treatment imaging follow-up with CT, MRI, or PET to assess for local control. Local tumor failure was defined as recurrence of tumor at treatment site as determined by enlarged lesion on CT, MRI, or PET scan and/or interpretation of the radiologist and tumor board members.

### Univariate and multivariate analysis of effects on survival

For each patient, the pretreatment CBC parameters within 3 months of SBRT was averaged and included with averaged values with other clinically relevant variables such as concurrent chemotherapy, Karnofsky Performance Status (KPS), and age in univariate Cox regression models to analyze their impact on overall survival. Overall survival was defined as time to death or last follow up from first treatment date. A selection criterion of *p* < 0.15 from the univariate model was used to determine the variables for the multivariate model. This was done because a small number of covariates was more appropriate for the small population size. Age, mean biologic equivalent dose across treatments, KPS, and NTLR were selected for the multivariate model.

A smoothed time-dependent ROC curve calibrated to 24 months of survival was used to generate an optimal cut point for NTLR^32^. Each patient’s NTLR was the average of their NTLR values prior to each SBRT treatment session. Optimal cut point to stratify patients into high and low risk groups was determined by the NTLR that maximized the Youden index, defined as the sum of sensitivity and specificity minus one. The Kaplan Meier method was used to calculate rates of overall survival. We compared the overall survival for these two groups using the log-rank test. All data were stored in a secure RedCap registry, and all analyses were performed in R (version 3.6.3)^33^.

## Results

In total, there were 42 patients with 138 lesions that met criteria for the study as detailed in Table 1. The median age at diagnosis was 21 years with a range of 4 to 47 years. The median pretreatment NTLR for the cohort was 5.3. Twenty-five out of 42 patients had confirmed death, and the median clinical follow up time for surviving patients was 24 months and median imaging follow up time was 7.7 months.

**Table 1:** Patient characteristics. Data is presented as either median [minimum to maximum] or as number in category (percent of whole group).

All patients (n = 42)	
Age at diagnosis (years)	21.5 [4.1, 47.7]
Male sex (%)	24 (57.1)
Mean pretreatment NTLR	5.26 [0.46, 29.19]
NTLR risk category = low (%)	24 (61.5)
Mean pretreatment KPS (%)	
<= 70	6 (15.0)
70 – 80	13 (32.5)
80 – 90	17 (42.5)
90 – 100	4 (10.0)
SBRT treatment courses	2 [1, 20]
Follow up time (months)	23.8 [6.0, 35.0]

*NLTR* neutrophil-to-lymphocyte ratio; *SBRT* stereotactic body radiotherapy.

**Figure 1:**
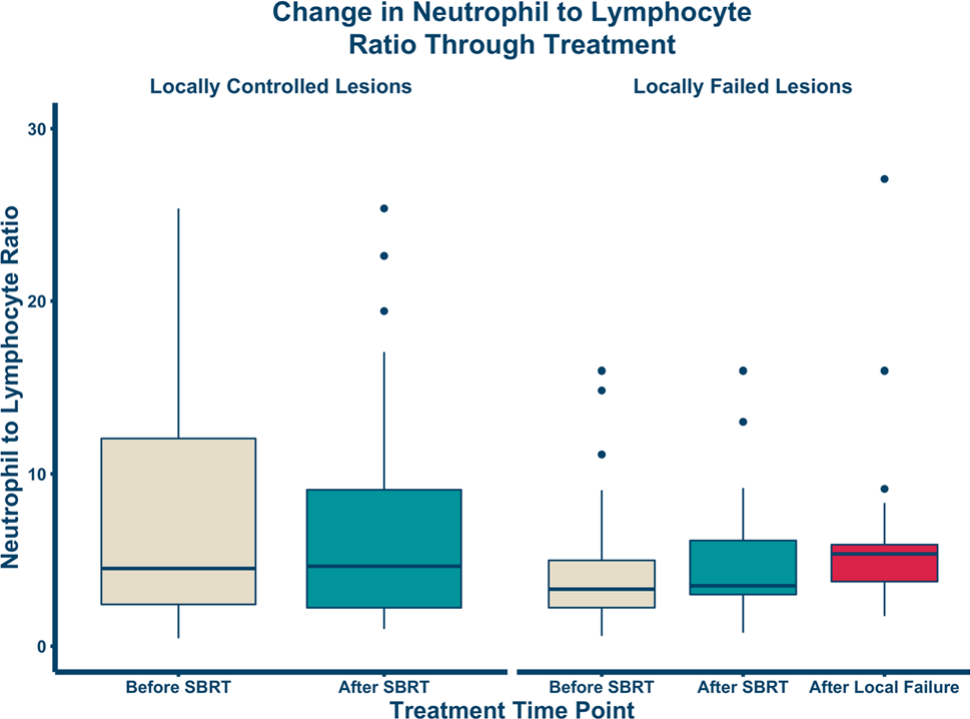
Change in neutrophil-to-lymphocyte ratio through SBRT. All data points are within 3 months of the noted time point. The change in median NTLR within the lesion controlled group was not significant (*p* = 0.30, N = 104) but was significant within the lesion failure group (*p* = 0.027; N = 34).

Details about all individual lesions (n = 138) were recorded in our registry (Table 2). Most sarcomas treated with SBRT were osteosarcoma or Ewing sarcoma (84/138), and the remaining were soft tissue sarcomas. Lesions received 22.2–140 Gy with a median dose of 40 Gy (most common regimen was 8 Gy × 5 fractions) with biological equivalent dose from 66.7 Gy to 419 Gy with a median dose of 117 Gy, using an alpha/beta ratio of 3. Most patients receiving SBRT were also receiving concurrent systemic therapy (79/138, 57%). A total of 34/138 (25%) lesions experienced local recurrence. Figure 1 demonstrates the change in neutrophil to lymphocyte ratio over time. Median NTLR for sarcoma patients with controlled lesions before and after SBRT were very similar: 5.0 (IQR: 2.7 – 13.4) and 4.8 (IQR: 2.3 – 9.4), respectively. In patients experiencing local recurrence, the median NTLR for before and after SBRT was lower [3.3 (IQR: 2.2–5.0) and 3.5 (IQR: 3.0–6.1), respectively], and at time of local failure was higher [5.6 (IQR: 3.8 – 5.9)]. For locally controlled lesions, there was no significant difference in NTLR between the before and after SBRT groups (*p* = 0.30). For lesions that experienced recurrence, there was a significant difference in NTLR amongst the three groups (before, after SBRT and at recurrence: *p* = 0.027).

**Table 2:** Descriptive characteristics of sarcoma lesions treated with SBRT.

All Lesions (n = 138) Median [min–max]	
Sarcoma histology (%)	
Clear cell carcinoma	3 (2.2)
Desmoplastic small round cell tumor	21 (15.3)
Ewing sarcoma	30 (21.9)
Osteosarcoma	54 (39.4)
Paraganglioma	7 (5.1)
Rhabdomyosarcoma	7 (5.1)
Small round blue cell sarcoma	4 (2.9)
Synovial sarcoma	11 (8.0)
Biological equivalent dose (Gy)	116.70 [66.7–419.3]
Pretreatment white blood cell count (10^3^/uL)^1^	5.10 [0.09–15.8]
Pretreatment hemoglobin (g/dL)	12.1 [7.7, 16.8-]
Pretreatment hematocrit (%)	35.6 [23.8–49.3]
Pretreatment platelets (10^3^/uL)	183[11–570]
Pretreatment absolute neutrophil count (10^3^/uL)	3.2 [0.6–14.5]
Pretreatment absolute lymphocyte count (10^3^/uL)	0.60 [0.08–3.8]
Pretreatment lactate dehydrogenase units/L)	211 [125–671]
Pretreatment albumin (g/dL)	4.20 [2.7, 7.8]
Pretreatment neutrophil to lymphocyte ratio	5.3 [0.46, 29.19]
Received concurrent therapy (%)^2^	79 (57.2)
Surveillance follow up time (months)	7.7 [0.5–35]
Known local recurrences (%)^3^	34 (28.8)

^1^Pretreatment complete blood count (CBC) data was charted within 3 months of treating the lesion.

^2^Concurrent therapy includes any chemotherapy or immunotherapy received at time of treatment.

^3^Local recurrence was determined by CT, MRI, or PET imaging surveillance. Data is presented as either median [min to max] or as number in category (percent of whole group).

Table 3 Cox proportional hazards regression analyses for overall survival in our patient population. Higher mean radiotherapy BED (HR: 0.99; 95% CI: 0.98 to 1.00) and higher patient KPS (HR: 0.91; 95% CI: 0.87 to 0.96) were significant protective factors for overall survival whereas higher average pretreatment neutrophil count (HR: 1.24; 95% CI: 1.06 to 1.45) and higher average pretreatment NTLR (HR: 1.11; 95% CI: 1.04 to 1.19) were significantly associated with poorer survival in the univariate model. In the multivariable model, only worse KPS (HR: 0.91; 95% CI: 0.86 to 0.96) and higher average pretreatment NTLR (HR: 1.14; 95% CI: 1.05 to 1.24) were significantly associated with overall survival.

**Table 3:** Cox proportional hazards analysis of clinical and treatment parameters associated with survival. Average KPS across treatments and pretreatment Neutrophil to Lymphocyte Ratio (NTLR) was the only variable associated with survival in both univariate and multivariate models.

Variable	UV Model HR	*p* value	MV Model HR	*p* value
Age	0.959 (0.917 to 1)	0.0629	0.952 (0.896 to 1.01)	0.104
Male sex	1.49 (0.657 to 3.37)	0.341		
Number of SBRT treatments	1.04 (0.964 to 1.13)	0.3		
Mean treatment BED	0.987 (0.976 to 0.998)	0.017	0.99 (0.978 to 1)	0.106
Mean KPS	0.911 (0.868 to 0.957)	0.00018	0.912 (0.864 to 0.963)	0.00083
Mean pretreatment neutrophil count	1.24 (1.06 to 1.45)	0.006		
Mean pretreatment lymphocyte count	0.637 (0.312 to 1.3)	0.216		
Mean pretreatment NTLR	1.11 (1.04 to 1.19)	0.003	1.14 (1.05 to 1.24)	0.0023
Received concurrent therapy	1.87 (0.742 to 4.69)	0.185		

*NLTR* Neutrophil-to-lymphocyte ratio; *SBRT* stereotactic body radiotherapy; *BED* biologic equivalent dose; *KPS* Karnofsky performance status.

**Figure 2:**
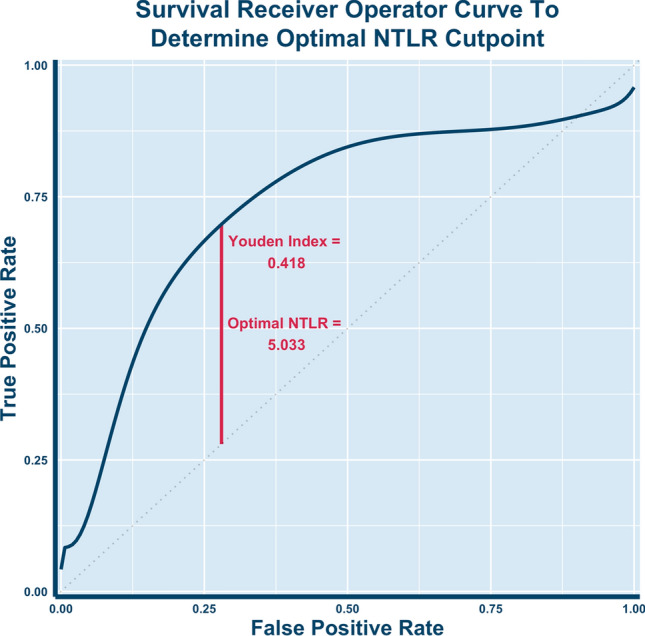
Receiver operating curve (ROC) for overall survival. The ROC was calibrated for 24 months of survival, and the optimal cut point that maximized sensitivity and specificity was a pre-SBRT NTLR value of 5.033. The Youden Index of .418 was calculated as [sensitivity (0.698) + specificity (0.720)] – 1.

**Figure 3:**
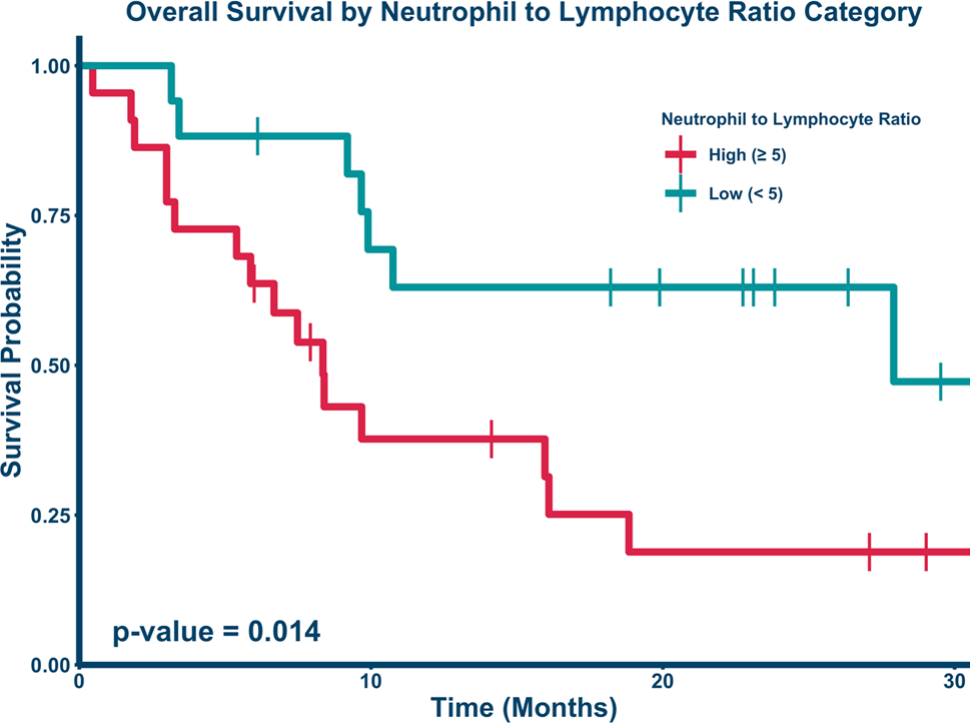
Kaplan–Meier curve comparing overall survival by NTLR ratio. Patients with an NTLR above 5 (red bottom line, n = 22) had a 1-year survival of 37.7% (95% CI: 21.4%–66.3%), while patients with an NTLR below 5 (teal, top line; n = 17) had a significantly improved 1- year survival of 63% (95% CI: 43.3%–91.6%, *p* = 0.014). 2-year survival was reduced to 18.8% (95% CI: 7.08% to 50.2%) for the high NTLR group and remained unchanged at 63% (95% CI: 43.3%–91.6%, *p* = 0.014) for the low NTLR group.

Fig. 2 demonstrates the time-dependent ROC curve calibrated to 24 months drawn to assess the optimal cutoff for stratifying high versus low risk groups based on NTLR. The optimal pre-SBRT NTLR cut point value was determined to be 5.033, which was rounded down to 5 and used for stratification into high risk and low risk groups. 24 patients (57%) were in the low risk NTLR category (< 5). Kaplan–Meier estimates for overall survival were calculated as shown in Fig. 3. Patients with a high NTLR (≥ 5) had significantly worse survival (*p* = 0.014). Patients with an NTLR ≥ 5 had a 1-year survival of 37.7% (95% CI: 21.4%–66.3%), while patients with an NTLR < 5 had a 1-year survival of 63% (95% CI: 43.3%–91.6%). 1-year survival in the entire cohort was 47.6% (95% CI: 34.3%–66.1%).

## Discussion

Our study demonstrated for the first time a significant correlation between increased NTLR and poor local control and significantly worse overall survival in high-risk metastatic sarcoma patients undergoing SBRT. These findings are consistent with the prior observations in non-small cell lung cancer^34^. Interestingly, patients with lesions with more durable local control after SBRT correlated with a higher baseline NTLR compared to those with lesions that had local failure. However, NTLR in those with lesions that were locally controlled remained stable after therapy and survival seemed to plateau. Among patients experiencing local failure, the NTLR increased slightly after SBRT and then increased significantly after confirmed sarcoma recurrence. These findings suggest that a rising NTLR may be a more sensitive biometric of impending recurrence than absolute elevation of baseline NTLR.

However, absolute NTLR was associated with a significantly worse survival outcome in patients with metastatic sarcoma treated with SBRT. High pretreatment NTLR was associated with poor survival in the multivariable Cox proportional hazards model controlled for age, dose, and KPS. We also calculated a cutoff NTLR value of 5 to risk-stratify patients with metastatic sarcoma into high risk and low risk groups. An elevated NTLR could reflect either inflammation associated with advancing disease, impaired lymphocyte numbers or resilience (i.e. associated with a low ANC); either of these immune parameters would be expected to affect the tumor inhibitory microenvironment and be associated with local failure^15^.

In oropharyngeal cancer, an association between loco-regional recurrence and elevated NTLR has been observed^35^. In the setting of early stage NSCLC patients treated with SBRT, an elevated NTLR predicted for worse survival, but there was no correlation with local control^34^. This study did not assess changes in NTLR over time, but only evaluated pretreatment variables collected within 2 months of SBRT. Our analysis did not yield a statistical association between time to local recurrence and NTLR prior to treatment in a Kaplan–Meier model; however, our findings may be limited by the sample size of our study.

NTLR may become a useful biometric to study during immunotherapy with radiation, especially SBRT. The potential synergy between radiotherapy and immunotherapy is a growing area of research interest^15^. While radiation can result in temporary immunosuppression, it can assist the immune system long term by reducing tumor burden via direct cytotoxicity and exposing neoantigens to the immune system. The induction of neoantigens by radiotherapy and subsequent immunization against those neoantigens have been demonstrated in prostate cancer^36^. SBRT in particular has been demonstrated to stimulate the cellular immune response through enhanced T cell activity, increased antigen presentation, and release of inflammatory cytokines^37^. SBRT mediated damage of one tumor site can lead to systemic activation of the immune system against neoantigens present in other tumor sites leading to an abscopal effect, which has been demonstrated in some clinical settings^37^.

An elevated neutrophil count can be observed in chronic inflammatory conditions, including cancer. Tumor production of cytokines such as IL-1 and IL-6 can induce neutrophilia, with higher circulating neutrophil counts at baseline theoretically corresponding with an increased burden of disease^38^. It has also been shown that increased absolute neutrophil count (ANC) has the potential to suppress cytotoxic T lymphocyte function^39^. The relative lymphopenia associated with high NTLR may be associated with a poorer host immune response against tumor antigens and thus a poorer prognosis. T cells are necessary for checkpoint inhibition therapies and the anti-cancer cellular therapies using CAR-T and CAR-NK^40^. Taken together, an elevated NTLR suggests an increased burden of disease and an impaired immune response, both of which are poor prognostic factors. In the context of cancer therapy, we believe NTLR is a relevant biomarker for assessing potential immune responsiveness following radiotherapy and a useful metric for risk-stratification.

Our study has several limitations. Since this study is retrospective, it is difficult to ascertain the time course of exactly when SBRT alters the NTLR biometric. Given the disease rarity and relatively recent implementation of SBRT for treatment of metastatic sarcoma, the small sample size of the cohort still represents a large series of SBRT treated lesions^11^. Additionally, most sarcoma patients undergoing SBRT were heavily pre-treated with systemic therapies, a factor that likely affected the bone marrow and circulating lymphocytes. Although treatments such as granulocyte colony stimulating factor (G-CSF) could confound results, no one in our SBRT cohort received concurrent G-CSF during or immediately after SBRT.

In conclusion, high NTLR at the time of SBRT treatment was prognostic for significantly worse overall survival of patients undergoing SBRT for metastatic sarcoma. A rise in NTLR was also seen at time of local progression. The NTLR biometric may have implications for future sarcoma treatment, pre-and post-SBRT sarcoma surveillance, and future sarcoma and SBRT clinical trial design warranting further investigation.

## Data Availability

Anonymized datasets recorded and analyzed during the current study are available from the corresponding author on reasonable request.

## Abbreviations

SBRT: Stereotactic Body Radiation Therapy
NTLR: Neutrophil to Lymphocyte Ratio
CBC: Complete Blood Count
KM: Kaplan–Meier
HR: Hazard Ratio
KPS: Karnofsky Performance Status
BED: Biological Effective Dose
UV: Univariate
MR: Multivariate
